# A cross-sectional study on the endorsement of reporting guidelines and clinical trial registration among immunology and allergy journals

**DOI:** 10.1371/journal.pone.0322003

**Published:** 2025-05-07

**Authors:** Adam Khan, Tim Smith, Asaad Chaudhry, Caleb A. Smith, Danya Nees, Griffin Hughes, Kaylyn Rowsey, Matt Vassar

**Affiliations:** 1 Office of Medical Student Research, Oklahoma State University Center for Health Sciences, Tulsa, Oklahoma, United States of America; 2 Department of Anesthesiology, University of Kansas Medical Center, Kansas City, Kanas, United States of America; 3 The Neurology Residency Program, University of Pennsylvania, Philadelphia, Pennsylvania, United States of America; 4 Department of Psychiatry and Behavioral Sciences, Oklahoma State University Center for Health Sciences, Tulsa, Oklahoma, United States of America; University of Liege: Universite de Liege, BELGIUM

## Abstract

**Background:**

Healthcare practitioners rely on research based on solid evidence for their clinical decisions, ensuring the provision of safe and effective patient care. The use of reporting guidelines and the registration of clinical trials enhance the reliability and credibility of research findings by promoting transparency and minimizing potential biases. However, it remains uncertain to what extent leading immunology and allergy journals have embraced these tools. This study aims to evaluate how commonly reporting guidelines and clinical trial registration are required and endorsed within leading immunology and allergy journals.

**Methods:**

We identified the top 100 journals in the subcategory of “Immunology and Allergy” using the Scopus CiteScore tool for the year 2021. We thoroughly reviewed the “Instructions for Authors” section of each journal, focusing on indications related to specific reporting guidelines as outlined by the Enhancing the Quality and Transparency of Health Research (EQUATOR) Network, as well as the practice of clinical trial registration. Our documentation categorized statements as “Not Mentioned,” “Recommended,” “Not Accepted,” or “Required.” The category “Not Accepted” specifically indicated that the journal explicitly did not accept the study designs associated with certain reporting guidelines, rather than implying bias against these guidelines. ensure equitable evaluation, we communicated with each journal to confirm the types of articles they accepted.

**Results:**

Among the 100 journals assessed, the CONSORT guideline emerged as the most frequently cited, with 60 journals recommending adherence and 13 requiring it. Conversely, the QUOROM guideline was the least commonly cited, with merely two journals recommending its adherence and none requiring it. Nineteen journals did not reference a single reporting guideline. Remarkably, clinical trial registration was required by 42 journals and recommended by 34.

**Conclusion:**

This study reveals variation in the adoption of reporting guidelines and clinical trial registration in immunology and allergy journals. While some journals strongly advocate for or require these practices, others do not emphasize them at all. This inconsistency affects research rigor and reproducibility, highlighting the need for stricter enforcement. Editors should encourage these practices to enhance transparency and minimize biases.

## Introduction

Allergic conditions are commonplace, and their prevalence has been increasing globally over the past few decades [1]. In order to better understand and treat allergic conditions — such as asthma, allergic rhinitis, and atopic dermatitis— high-quality research is paramount. As systematic reviews become increasingly published within the field of immunology and allergy [2], it is imperative to have a clear and standardized framework for reporting to ensure research is conducted and presented rigorously. Reporting guidelines serve as these frameworks by providing a structured checklists for authors to follow, promoting transparency, consistency, and methodological rigor [3–5]. While reporting guidelines alone do not directly address risk of bias, they help ensure that key aspects of study design, methodology, and analysis are thoroughly documented, which aids in the subsequent assessment of bias [4,6]. Additionally, clinical trial registration ensures that studies are pre-registered, reducing the risk of selective reporting and further enhancing transparency of study outcomes. Together, these tools contribute to more reliable, reproducible research and provide a foundation for quality systematic reviews. Immunology and allergy authors may find relevant reporting guidelines within the online library of the Enhancing the Quality of Transparency of Health Research (EQUATOR) Network.

The EQUATOR Network has been central to the deposition of more than 500 guidelines commonly used for popular study designs [7,8]. These well-studied reporting guidelines have been shown to improve the quality of studies in most popular study designs used in medical literature. For example, the Consolidated Standards of Reporting Trials (CONSORT) guideline has proven to enhance the quality of data reporting and reproducibility of randomized control trials (RCTs) [4,9]. Likewise, the Preferred Reporting Items for Systematic Reviews and Meta-Analyses (PRISMA) guideline has shown similar results for systematic reviews/meta-analyses [10,11]. Although important, previous studies in immunology and allergy reveal that guidelines are often not applied. When used, they are not comprehensively followed, highlighting the need for improved implementation and dissemination of these tools [12,13].

One approach immunology and allergy journals can take to improve the implementation of reporting guidelines is requiring or recommending their use in the “instructions to authors” webpage for investigators wishing to publish their research. Agha *et al*. found the reporting quality of research significantly improved when journals required the implementation of CONSORT, Strengthening the Reporting of Observational Studies in Epidemiology (STROBE), PRISMA, and Standards of reporting of Diagnostic Accuracy (STARD) guidelines for their authors [14]. Other studies regarding adherence to common guidelines exhibit a similar trend as well [15,16]. However, despite the reporting improvements these guidelines make in regards to the quality of research, some academic journals do not consistently require or even recommend their use [16–20].

The use of public registries for clinical trials and systematic reviews is an established approach to promote accountability in research practices. Clinical trial registration is supported by the World Health Organization (WHO) due to evidence indicating these registries help mitigate publication bias and enhance research integrity by holding investigators accountable for reporting their predefined outcomes [21]. Additionally, The International Committee of Medical Journal Editors (ICMJE) actually requires RCTs published within their network to be registered prior to patient enrollment [22,23]. While registration itself does not guarantee improved reporting, it sets a standard for investigators to adhere to their original protocols, reducing the likelihood of selective reporting or post hoc changes. Studies that are registered prior to commencement have shown an improved transparency of results and reduced reporting bias [24–26]. Therefore, enforcing study registration could contribute to advancing high-quality research practices. Journals may uphold the use of clinical trial registrations by recommending or requiring their use in the “instructions to authors” webpage. However, this practice is not observed in many fields of medicine [27–29].

Evidence suggests reporting guidelines and study registration enhance the methodological rigor and quality of research. However, it remains unclear how well these tools are enforced or recommended by journals publishing immunology and allergy research; to our knowledge, there is a lack of research investigating this topic. Therefore, the purpose of this study was to investigate the frequencies at which reporting guidelines and clinical trial registration are required or recommended by top immunology and allergy journals.

## Methods

### Study design

We conducted a cross-sectional analysis in accordance with the Strengthening the Reporting of Observational Studies in Epidemiology (STROBE) checklist [30]. Data were extracted from the “instructions to authors” page of identified immunology and allergy journals.

### Search strategy

One investigator (CAS), alongside a medical research librarian, identified eligible journals for this study by using the 2021 Scopus CiteScore tool [31]. The Scopus subcategory used was “Immunology and Allergy.” Google Scholar Metrics h5-index was used to affirm the top-twenty journals identified by Scopus to ensure accuracy [32].

### Eligibility

The top 100 peer-reviewed clinical journals within the “Immunology and Allergy” subcategory identified by the 2021 Scopus CiteScore tool were assessed. Journal websites whose “instructions to authors” were written in a language other than English were included in our study and were translated using ‘Google Translate’ [33,34].

### Exclusion criteria

**Fig 1:**
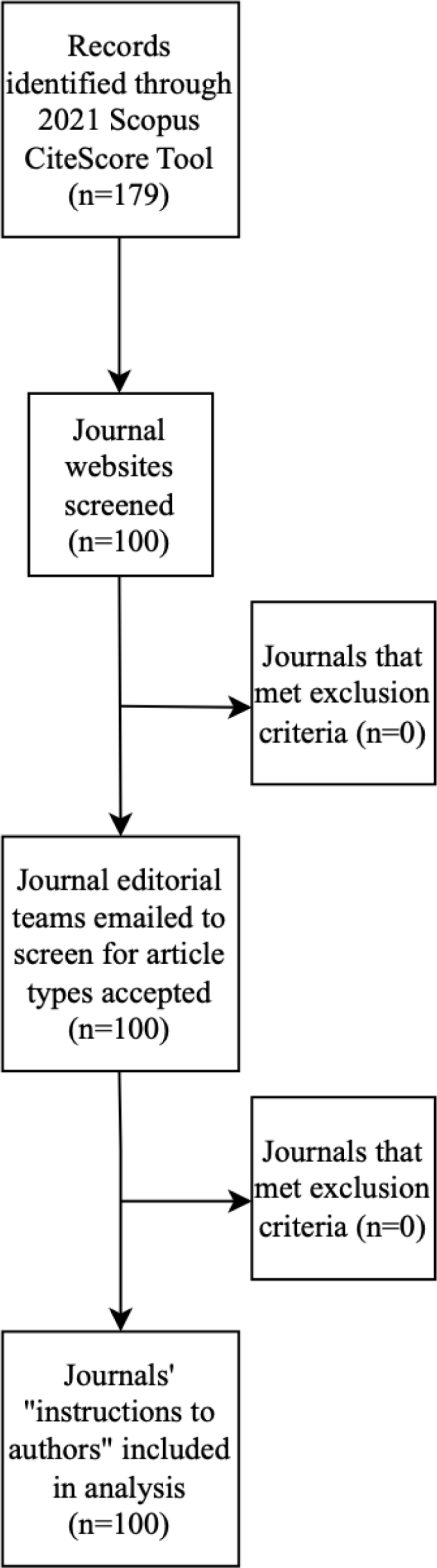
PRISMA flow diagram of journal selection process.

Journals were excluded from our study sample if they had been discontinued, to ensure that the analysis focused solely on active journals still relevant for publication purposes. Journals were also excluded if the journal’s web page lacked any sort of communication capabilities with the editorial office. This was done to prevent the unfair assessment of journals without at least giving the editors the opportunity to elaborate on the journal’s publication policies. Finally, academic books were excluded as they offer a summary on current research in the field rather than publishing original research. If a journal was excluded, a subsequent journal identified by the Scopus CiteScore summary was used to maintain a sample size of 100 (Fig 1).

### Data collection process

Two investigators (AK, TS) extracted data from the “instructions to authors” page of the included journals in a masked, duplicate fashion. This was accomplished by using a standardized Google Form designed *a priori* by investigators CAS, DN, and MV. After data extraction, the two investigators were unmasked to reconcile one another’s data. Discrepancies in collected data were resolved by consensus between the two investigators (AK, TS). In the event a disagreement persisted past discussion, CAS was referred to for resolution.

### Data items

The following data items were extracted for each journal included in the study: email responses of journal editors, journal title, five-year impact factor, mention of the EQUATOR Network in the “instructions to authors,” mention of the ICMJE in the “instruction to authors,” and geographical zone of publication. For each journal’s “instructions to authors’’, we extracted statements regarding specific reporting guidelines used in popular study designs. Descriptions of these reporting guidelines and their respective study designs are found in Table 1. Additionally, statements regarding study registration at databases including: Clinicaltrials.gov, WHO International Clinical Trial Registry Platform, PROSPERO, or any other trial and database were extracted.

**Table 1:** Reporting guidelines and study designs.

Study design	Respective reporting guideline
Animal Research	ARRIVE
Case Reports	CARE
Clincal Trials	CONSORT
Clinical Trial Protocols	SPIRIT
Diagnostic Accuracy	STARD
	TRIPOD
Economic Evaluations	CHEERS
Observational Studies in Epidemiology	MOOSE
	STROBE
Qualitative Research	COREQ
	SRQR
Quality Improvement	SQUIRE
Systematic Reviews and Meta-Analyses	PRISMA
	QUOROM
Systematic Review and Meta-Analysis Protocols	PRISMA-P

ARRIVE, Animal Research: Reporting of In Vivo Experiments; CARE, Case Reports guidelines checklist; CHEERS, Consolidated Health Economic Evaluation Reporting Standards; CONSORT, Consolidated Standards of Reporting Trials; COREQ, Consolidated Criteria for Reporting Qualitative Research; MOOSE, Meta-Analysis of Observational Studies in Epidemiology; PRISMA, Preferred Reporting Items for Systematic Reviews and Meta-Analyses; PRISMA-P, Preferred Reporting Items for Systematic Review and Meta-Analysis Protocols; QUOROM, Quality of Reporting of Meta-analyses; SPIRIT, Standard Protocol Items: Recommendations for Interventional Trials; SQUIRE, Standards for Quality Improvement Reporting Excellence; SRQR, Standards for Reporting Qualitative Research; STARD, Standards for Reporting Diagnostic Accuracy Studies; STROBE, Strengthening the Reporting of Observational Studies in Epidemiology; TRIPOD, Transparent Reporting of a Multivariate Prediction Model for Individual Prognosis or Diagnosis.

For each identified journal, it was determined if a given guideline or study registry was required, recommended, not required, or not mentioned. Language in the “instructions to authors” such as “required,” “must,” “need,” “mandatory,” and “studies will not be considered for publication unless…” was interpreted as “required.” Language such as “recommended,” “encouraged,” “should,” and “preferred” was interpreted as “recommended.” In the case language was unclear, a decision was made by consensus between study investigators. If a journal referred readers directly to the EQUATOR Network website for the requirements or recommendations, it was assumed the relevant guideline for each given article type was being considered.

To prevent the assessment of a journal based on a reporting guideline of a study design not accepted by the journal, the editorial team of each identified journal was contacted by email to inquire about accepted articles types as seen in Table 1. Two investigators (AK, TS) corresponded with editorial teams by emailing once a week for three consecutive weeks to improve response rates [35]. A standardized email prompt designed by investigator CAS was used to minimize variability in communication efforts. If no email response was received after three weeks, no judgements were made regarding accepted article types and the given journal Instructions for Authors was scrutinized against all data items being assessed.

### Investigator training

Prior to beginning the study, investigator CAS discussed the scope, methods, and rationale as well as provided instructions to the two investigators (AK, TS) for the data collection process. Before the induction of the study, the two investigators (AK, TS) were also required to extract data from five journals not included in the study sample in a masked, duplicate manner as practice. After doing this, the two investigators convened and discussed any discrepancies in extracted data. Investigators CAS and DN provided further instructions and another set of five journals to extract from if needed. The two investigators (AK, TS) repeated this process until no discrepancies were obtained in their practice sample. Only once this training was complete were the two investigators (AK, TS) allowed to extract data from the full study sample.

### Outcomes

The primary outcome of this study was the proportion of journals’ “instructions to authors” that required/recommended the use of the reporting guidelines found in Table 1. The secondary outcome was the proportion of journals that required/recommended the registration of clinical trials.

### Data synthesis

Using R (version 4.2.1) and RStudio, we reported descriptive statistics for immunology and allergy journal reporting guidelines. The descriptive statistics covered two main aspects: (1) frequencies and percentages of guidelines used in the included journals, and (2) the proportion of journals requiring clinical trial registration. Since our analysis concentrated on the instructions provided to authors at the journal level, rather than evaluating individual studies, we did not perform bias assessments, as these are typically conducted at the study level.

### Reproducibility

Our study follows a pre-established protocol designed *a priori*. To ensure transparency and promote study reproducibility, we uploaded this protocol, along with raw data, analysis scripts, extraction forms, and standardized email prompts, on the Open Science Framework (OSF) (https://osf.io/wrtke/) [36].

## Results

### Journal characteristics

In total, our findings encompassed an evaluation of 100 journals that met our predefined exclusion criteria. Detailed information about the 100 included journals is provided in Table 2.

**Table 2:** Editorial policies of included journals.

Journal	5-YR impact factor	Region	Equator network	ICMJE
Nature Reviews Immunology	91.77	Europe	Yes	Yes
Immunity	39.54	North America	No	No
The Lancet Rheumatology	35.54	Europe	Yes	Yes
Annual Review of Immunology	35.20	North America	No	Yes
Nature Immunology	31.00	Europe	Yes	Yes
Science immunology	25.30	North America	Yes	Yes
Trends in Immunology	21.10	Europe	No	No
Annals of the Rheumatic Diseases	20.70	Europe	Yes	Yes
Cellular and Molecular Immunology	17.70	Asia	Yes	Yes
Journal of Experimental Medicine	16.42	North America	Yes	Yes
Immunological Reviews	16.06	North America	Yes	Yes
Seminars in Immunopathology	14.33	Europe	Yes	Yes
Journal for ImmunoTherapy of Cancer	13.89	Europe	Yes	Yes
Journal of Allergy and Clinical Immunology	13.77	North America	No	Yes
Seminars in Immunology	12.92	Europe	No	Yes
Arthritis and Rheumatology	12.68	North America	No	Yes
Cytokine and Growth Factor Reviews	12.32	Europe	No	No
Autoimmunity Reviews	11.77	Europe	No	No
Journal of Autoimmunity	10.52	Europe	No	Yes
Clinical Reviews in Allergy and Immunology	9.96	North America	Yes	Yes
Journal of Allergy and Clinical Immunology: In Practice	9.92	Europe	No	Yes
JHEP Reports	9.92	Europe	Yes	Yes
Mucosal Immunology	9.44	Europe	Yes	Yes
Frontiers in Immunology	8.88	Europe	No	Yes
Inflammation and Regeneration	8.39	Europe	Yes	Yes
OncoImmunology	8.24	North America	Yes	Yes
Current Opinion in Immunology	8.22	Europe	No	Yes
American Journal of Transplantation	8.04	North America	Yes	Yes
Journal of Clinical Immunology	7.42	North America	Yes	Yes
Advances in Immunology	7.40	North America	No	No
Journal of Innate Immunity	7.36	North America	Yes	Yes
Immunology	7.20	North America	Yes	Yes
Clinical and Translational Immunology	7.03	Europe	Yes	No
Journal of Investigational Allergology and Clinical Immunology	6.99	Europe	No	Yes
Cancer Immunology, Immunotherapy	6.69	North America	Yes	Yes
Inflammatory Bowel Diseases	6.59	North America	No	Yes
Clinical Immunology	6.50	North America	No	No
Journal of Infectious Diseases	6.50	North America	Yes	Yes
International Immunology	6.31	Europe	No	No
Journal of Immunology	6.17	North America	No	Yes
European Journal of Immunology	6.09	North America	Yes	Yes
Allergology International	6.05	Asia	No	Yes
Immune Network	6.04	Asia	No	Yes
Arthritis Research and Therapy	5.99	Europe	Yes	Yes
mAbs	5.94	North America	Yes	Yes
Journal of Leukocyte Biology	5.85	North America	No	Yes
Journal of Microbiology, Immunology and Infection	5.85	Asia	No	Yes
Journal of NeuroImmune Pharmacology	5.81	North America	Yes	Yes
Cytotherapy	5.79	Europe	No	Yes
Annals of Allergy, Asthma and Immunology	5.67	North America	No	Yes
Pediatric Allergy and Immunology	5.65	North America	Yes	No
International Reviews of Immunology	5.63	Europe	Yes	Yes
Journal of Immunology Research	5.60	Europe	No	No
Clinical and Experimental Allergy	5.60	North America	Yes	Yes
International Immunopharmacology	5.60	Europe	No	Yes
World Allergy Organization Journal	5.56	Europe	No	Yes
RMD Open	5.27	Europe	Yes	Yes
Clinical and Experimental Immunology	5.16	Europe	No	Yes
Allergy, Asthma and Immunology Research	5.12	Asia	No	Yes
Current Allergy and Asthma Reports	5.11	North America	Yes	Yes
Clinical and Translational Allergy	5.05	North America	Yes	Yes
Current Topics in Microbiology and Immunology	5.03	Europe	Yes	Yes
Immunology and Cell Biology	5.01	North America	Yes	No
Expert Review of Clinical Immunology	4.90	Europe	Yes	Yes
AIDS	4.76	North America	Yes	Yes
Journal of Rheumatology	4.76	North America	No	No
Journal of Immunotherapy	4.63	North America	Yes	Yes
International Forum of Allergy and Rhinology	4.52	North America	No	Yes
Human Vaccines and Immunotherapeutics	4.40	North America	Yes	Yes
Inflammation	4.36	North America	Yes	Yes
Archivum Immunologiae et Therapiae Experimentalis	4.22	Europe	Yes	Yes
Journal of Reproductive Immunology	4.14	Europe	Yes	Yes
Immunotherapy	4.14	Europe	No	Yes
Immunology Letters	4.13	Europe	No	Yes
Cytokine	4.03	Europe	No	Yes
American Journal of Reproductive Immunology	3.99	North America	Yes	Yes
Expert Review of Respiratory Medicine	3.93	Europe	Yes	Yes
Clinical and Experimental Rheumatology	3.92	Europe	No	Yes
Journal of Global Antimicrobial Resistance	3.92	Europe	No	Yes
Immunology and Allergy Clinics of North America	3.90	North America	No	Yes
Asian Pacific Journal of Allergy and Immunology	3.88	Asia	No	Yes
Pathogens and Disease	3.80	Europe	Yes	Yes
Ocular Immunology and Inflammation	3.68	North America	Yes	Yes
Transfusion Medicine and Hemotherapy	3.62	Europe	Yes	Yes
Medical Microbiology and Immunology	3.60	North America	Yes	Yes
Journal of Neuroimmunology	3.51	Europe	No	Yes
Immunobiology	3.37	Europe	No	Yes
APMIS	3.36	North America	No	No
International Archives of Allergy and Immunology	3.35	Europe	Yes	Yes
Rheumatology International	3.25	Europe	Yes	Yes
British Journal of Biomedical Science	2.15	Europe	No	Yes
Allergy: European Journal of Allergy and Clinical Immunology	N/A	Europe	Yes	Yes
Antibody Therapeutics	N/A	Europe	No	Yes
Biologics: Targets and Therapy	N/A	Other	Yes	Yes
Clinical and Molecular Allergy	N/A	Europe	Yes	Yes
Current Tropical Medicine Reports	N/A	North America	Yes	Yes
International Journal of Inflammation	N/A	North America	No	Yes
Pathogens and Immunity	N/A	Europe	No	Yes
Therapeutic Advances in Vaccines and Immunotherapy	N/A	Europe	Yes	Yes
Transplantation and Cellular Therapy	N/A	North America	No	Yes

The five-year impact factor of the included journals ranged from 2.15 to 91.77 (mean = 9.39; standard deviation = 11.27). Journals were predominantly sourced from European regions (50/100,), while the remaining journals originated from North America (43/100), Asia (6/100), and other global regions (1/100). After receiving correspondence from journal editors, specific reporting guideline data were excluded from proportion calculations if the editors indicated that the respective studies were not accepted. Instances of exclusions include: QUOROM (1/100), MOOSE and STROBE (1/100), SRQR (1/100), CHEERS (1/100), COREQ (1/100), CARE (3/100), SPIRIT (2/100), PRISMA-P (2/100), and ARRIVE (1/100). These exclusions are denoted by asterisks in Table 2.

### Reporting guidelines

**Fig 2:**
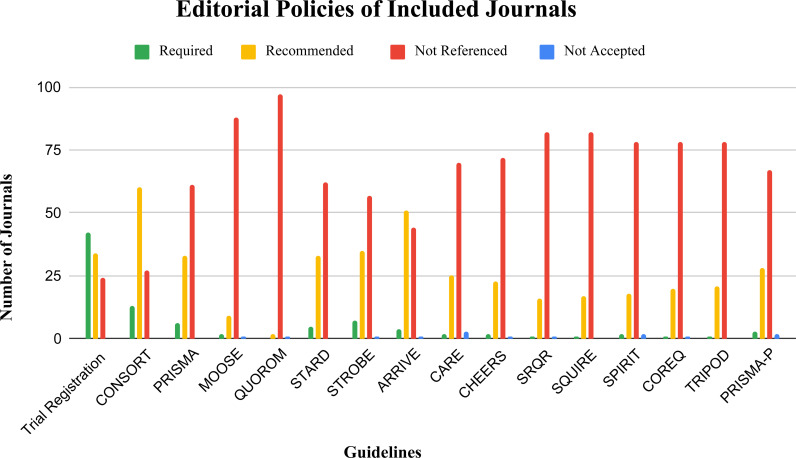
Editorial policies on reporting guidelines and clinical trial registration. “Not Accepted” indicated journals explicitly did not accept submissions of specific study designs.

Eighty-eight journals referenced the ICMJE guidelines (88/100), while 55 (55/100) referenced the EQUATOR Network. A total of 19 (19/100) journals did not refer to a single reporting guideline. Among the studied guidelines, CONSORT, ARRIVE, and STROBE were the most frequently referenced. CONSORT was referenced by 73 journals (73/100). Of these, 13 (13/73) required adherence. Secondly, ARRIVE was referenced by 55 journals (55/100) and specifically required by 4 (4/55). STROBE was referenced by 42 journals (42/100) and was required by 7 (7/42) of this subset. The least frequently referenced guidelines were QUOROM, MOOSE, and SQUIRE. A total of 97 journals (97/100) did not reference QUOROM. MOOSE was not referenced in 88 journals (88/100), while SQUIRE was not referenced in 82 journals (82/100) (Fig 2).

### Clinical trial registration

Of the 100 journals evaluated for clinical trial registration, forty-two (42/100) enforce clinical trial registration while thirty-four (34/100) recommended this practice. Twenty-four journals (24/100) failed to make any reference to clinical trial registration policies (Fig 2).

## Discussion

Our study found inconsistent endorsement of reporting guidelines among leading immunology and allergy journals with 81/100 recommending or requiring at least one reporting guideline. Among the 100 journals under examination, a total of 13 journals required the CONSORT guideline and 60 journals recommended it, making it the most frequently endorsed reporting guideline. In contrast, QUOROM was the least referenced guideline with only two journals recommending it and none requiring it. The limited uptake of the QUOROM guideline within our surveyed immunology and allergy journals can potentially be attributed to its replacement by the PRISMA guideline as the favored reporting measure for systematic reviews and meta-analyses [37]. Furthermore, of the 100 journals examined, 42% of the journals required clinical trial registration, and 34% recommended it. This represents progress in improving transparency and reproducibility in clinical research; however, gaps remain, as nearly a quarter of the sampled journals have yet to adopt the policies recommending or requiring clinical trial registration [S1 Table].

Among the journals included in our study, 13% required CONSORT, a major guideline for authors reporting clinical trials—making it the most frequently required guideline among those examined. This finding highlights the stronger emphasis placed on CONSORT in immunology and allergy journals compared to other fields of medicine. For example, Zuniga-Hernandez et al., found that in 82 endocrinology journals, less than half of the journals referenced endorsement of CONSORT, PRISMA, and STROBE [38]. Interestingly, this same study also found that journals that endorsed CONSORT were more likely to require clinical trial registration and vice versa. Our results are consistent with the latter finding, with immunology and allergy journals demonstrating relatively high endorsement rates for both CONSORT (73%) and clinical trial registration (76% when combining recommended and required policies). Further, Shantikumar et al. found, when examining 136 surgical journals, that only 29% of their sample endorsed CONSORT and 32% endorsed trial registration [39]. Because of the value clinical trials represent in the medical literature, further research on the relationship between reporting guideline endorsement and clinical trial registration should be considered. Clinical trials represent the primary method through which researchers ascertain the safety and efficacy of new treatments or preventive measures such as novel drugs, diets, or medical devices for individuals dealing with life-threatening illnesses or chronic health issues [40]. Poorly designed clinical trials risk the dissemination of inaccurate or biased results which pose considerable threats to evidence-based practice and human health as well as creating unnecessary financial waste [41]. Given the significance of robust clinical trials in clinical research, all journals should consider enforcing the adherence to CONSORT guidelines to be considered for publication. Enforcing CONSORT adherence would not only improve reporting transparency but also ensure that clinical trial findings are presented in a standardized and comprehensive manner. This would ultimately benefit evidence-based practice and patient care.

Less than half of the included journals explicitly required clinical trial registration and nearly a quarter failed to reference registration at all. Clinical trial registration policies can help reduce publication bias and selective reporting, improve transparency, and increase accountability for trialists [26]. In a 2005 effort to promote transparency and accountability in clinical research, the International Committee of Medical Journal Editors (ICMJE) made prospective clinical trial registration a requirement for publication in their member journals [42]. Nonetheless, even among the top-impact journals linked with US medical societies, adherence to the ICMJE’s registration policies remains sub-standard [43]. Beyond this, studies of other medical specialties have found similar results to ours. For example, a 2016 study on clinical trial registration policies in emergency medicine journals found that less than half of the included sample referenced trial registration expectations [44]. Even more similar to our results is data from a study on oncology journals that found approximately three-quarters of journals endorse trial registration [45]. Authors have the ability to maximize credibility and minimize selective reporting bias while improving access to information for clinicians and patients alike through prospective trial registration. Immunology and allergy journals therefore can play a pivotal role in improving research quality by enforcing proper registration practices.

Academic journals act as gatekeepers of disseminating unbiased and reputable research for the betterment of medicine. Consequently, stringent publication prerequisites, including adherence to reporting standards and clinical trial registration, are anticipated. However, many journals provide vague or unclear directives, creating challenges for researchers attempting to comply with publication requirements. For instance, we found that many journals lacked clarity on accepted study types, had “instructions to authors” webpages that were complex and difficult to navigate, and others did not reference EQUATOR or relevant reporting guidelines in their “instructions to authors” sections altogether. These aspects contribute to misunderstanding of journal expectations and inadequate reporting, undermining research transparency and reproducibility [46]. Therefore, clear and explicit journal policies are necessary to mitigate these problems. Evidence from a 2022 study examining surgical journals demonstrated that clear expectations for reporting guideline and clinical trial adherence was associated with higher impact factors or more total citations [47]. Ultimately, it may be in the best interest of journals to endorse reporting guidelines and clinical trial registration.

Importantly, this study did not analyze the actual publications published by these journals to determine if reporting guidelines were actually adhered to or if clinical trials were properly registered. It remains possible that even if a journal has policies in place, authors may not adhere to them. For instance, Hayes et al. found that in the top five general medical journals that endorse CONSORT, the average adherence rate by relevant articles was only 67% [48]. There has been disparity between journals’ public stance and practical action regarding adherence to guidelines for authors, which may mislead readers into assuming outcomes and other regulations are correctly reported [49]. Future studies could investigate the specifics of legitimate adherence to the journal guidelines that authors publish under. Specifically, research could be directed toward understanding the barriers and facilitators that contribute to the dissemination of reporting guideline policies and how they are communicated between journals and prospective authors.

### Strengths and limitations

Our study has important strengths. First, our data were extracted in a masked, duplicate fashion to minimize bias and reduce extraction errors, a method recommended by the Cochrane Collaboration [50]. Second, our study adhered to a pre-established protocol accessible to readers on OSF, enhancing both the transparency and reproducibility of our research. Lastly, this study is reported according to the STROBE guidelines for observational studies. Despite these strengths, this study is not without limitations. A potential constraint of our study pertained to the relatively low response rate to our email queries sent to editors. This could have led to disparities between our collected data and the actual publication policies of journals. Nevertheless, it should be noted that this discrepancy underscores the communication challenges researchers encounter while striving to publish in specific journals. Furthermore, the cross-sectional nature of our study restricts the extent to which our findings can be applied broadly, necessitating cautious interpretation of our results.

## Conclusion

This study found that academic journals in the field of immunology and allergy do not consistently promote the use of reporting guidelines or clinical trial registries. These findings have implications for the rigor and reproducibility of medical research, and suggest the need for greater standardization and enforcement of reporting guidelines and clinical trial registration requirements. We recommend that the editors of immunology and allergy journals encourage the use of reporting guidelines and clinical trial registration policies to improve transparency and mitigate the potential for biases.

## Supporting information

S1 Table Data set. (XLSX)
